# Vaccine Hesitancy in Children—A Call for Action

**DOI:** 10.3390/children3020007

**Published:** 2016-04-29

**Authors:** Annabelle de St. Maurice, Kathryn M. Edwards

**Affiliations:** Department of Pediatrics, Vanderbilt University, Nashville, TN 37232, USA; annabelledestmaurice@gmail.com

**Keywords:** immunization, refusal of vaccines, vaccine benefits and risks, health care providers education

## Abstract

Immunizations have made an enormous impact on the health of children by decreasing childhood morbidity and mortality from a variety of vaccine-preventable diseases worldwide. The eradication of polio from Nigeria and India is one of the most recent victories for one of the greatest technological advances in human history. Despite these international successes, the United States has experienced the re-emergence of measles, driven largely by increasing parental refusal of vaccines. Pediatricians should be trained to be very knowledgeable about vaccines and should continue to advocate for parents to immunize their children.

Immunizations have made an enormous impact on the health of children by decreasing childhood morbidity and mortality from a variety of vaccine-preventable diseases worldwide. The eradication of polio from Nigeria and India is one of the most recent victories for one of the greatest technological advances in human history. Despite these international successes, the United States has experienced the re-emergence of measles, driven largely by increasing parental refusal of vaccines [1,2]. While the overwhelming majority of parents adheres to the immunization schedule recommended by the Advisory Committee on Immunization Practices (ACIP), some parents choose to delay vaccines or refuse some vaccines altogether [3,4]. Parents cite concerns about the necessity, efficacy, and safety of vaccines [5,6,7,8]. Such concerns, regardless of whether or not they are supported by medical evidence, influence a parent’s decision-making process. Ongoing research has attempted to define the best methods to address parental concerns about vaccine hesitancy [9]. Although studies have had mixed results in determining the most effective strategies for countering vaccine hesitancy, numerous studies have demonstrated the important role of pediatricians in influencing parental vaccine decision making [6,10,11,12].

To be successful in discussing vaccine benefits and risks pediatricians and other health care providers must be well-informed and confident about the overwhelming benefits and safety of immunizations. However, some of these providers and pediatric resident trainees do not feel prepared or informed enough to discuss vaccines and vaccine safety with families [13,14]. A recent survey of pediatricians revealed that younger pediatricians may have more doubts about the safety of vaccines and concerns about vaccine efficacy [15]. Since most recent medical graduates and young physicians have not witnessed the potentially devastating consequences of infection with measles, varicella zoster or pertussis, they may underestimate the importance of immunizations. Although a basic knowledge about vaccine preventable diseases and vaccine adverse effects is required to pass the General Pediatrics certification exam, the American Board of Pediatrics does not require pediatricians to have a comprehensive understanding of vaccine development, testing and safety monitoring. Medical schools and residency programs should increase training regarding vaccines and vaccine-preventable diseases, with a focus on the comprehensive vaccine manufacturing and development process, as well as post-licensure monitoring, that occur in order to ensure vaccine safety.

Knowledge alone is not sufficient. In order to be effective immunization advocates, health care providers must confidently convey that parents should adhere to the immunization schedule approved by ACIP. Rather than asking parents if they plan to accept vaccines, pediatricians should take a directive role when discussing vaccines. We do not ask parents if they plan to use a car seat or place their infant on his or her back to sleep. A study by Opel, *et al.*, supports this more paternalistic approach [16]. Their study demonstrated that parents were more likely to refuse vaccines if immunizations were presented as optional. Although parents should have an active and important role in their child’s health, they should also be cognizant of evidence-based recommendations. Recent medical school graduates are taught to discuss medical matters in the context of “shared-decision making”, in which patients and physicians share responsibility for medical decisions [17]. Families, patients, and professional organizations such as the American Academy of Pediatrics endorse this less paternalistic approach [18], however the emphasis on shared decision making should not supplant the practice of evidence-based medicine [19]. Instead, pediatricians should integrate evidence-based medicine and knowledge about immunizations into discussions with families, while encouraging families to participate in the shared decision-making process by asking questions and voicing concerns. Physicians should be committed to these evidence-based recommendations and stress that deciding to vaccinate one’s child is the best decision that a parent can make.

Residency training programs should continue to emphasize immunizations and immunization safety within their curricula. In addition, residents should learn how to talk to parents about immunizations in an effective manner. In order to communicate effectively and build trust with parents, pediatricians and other health care providers, as a whole, must be better informed about the safety and efficacy of vaccines and the rigorous mechanisms that safeguard the vaccine development process.
